# Normal grief and its correlates in Lubumbashi, an urban city in the Democratic Republic of Congo

**DOI:** 10.11604/pamj.2016.24.24.5533

**Published:** 2016-05-06

**Authors:** Katabwa Kabongo Joe, Mala Ali Mapatano, Tshibangu Manyonga, Costa Kazadi Mwadianvita, Mutombo Valérien, Wembonyama Stanis, Mukendi Kavulu, Kashala Espérance

**Affiliations:** 1 Department of Internal Medicine, Psychiatry Unit, University of Lubumbashi, Democratic Republic of Congo; 2 Department of Public Health, University of Kinshasa (UNIKIN), Democratic Republic of Congo; 3 Department of Psychology, Excellence Centre, University of Lubumbashi, Democratic Republic of Congo; 4 Department de Science Biomedical University of Lubumbashi, Democratic Republic of Congo; 5 Department of Neurology, University of Mbuji Mayi, Democratic Republic of Congo; 6 Department of Paediatrics, University of Lubumbashi, Democratic Republic of Congo; 7 Neurology Unit, Mons Regional Hospital Complex and University Catholique de Louvain (UCL), Belgium, Department of Paediatrics, University of Lubumbashi, Democratic Republic of Congo; 8 Centre for International Health, University of Bergen, Norway

**Keywords:** Grief, depression, complications, anxiety, Democratic Republic of Congo

## Abstract

**Introduction:**

Grief is a universal experience faced at one time or another by most people during their lives. Response to grief and bereavement losses can lead to psychiatric disorders such as mood disorders, post traumatic responses, insomnia loss of appetite, anxiety, and depression. The aim of our study is to value in our community the physical and psychological complications of a normal grief.

**Methods:**

We conducted a cross-sectional study from March 2012 to September 2012 in Lubumbashi, Democratic Republic of Congo. Two questionnaires, the Hamilton Anxiety Scale and the Beck Depression Inventory scale were used as screening tool. A snow ball sampling method was performed and the questionnaires were administered only to those who consented to participate in the study.

**Results:**

A total of 78 subjects were included in the study of which 87.2% were aged between 14-50 years old. The majority of the subjects were female 65.4%, and about a quarter (28%) was unemployed. The main correlates of the grief reported in the present study were being treated as witchcraft or accused to be responsible of a death (68%), being rejected by family and not being allowed to inherit (32%). Being homeless was reported in 26%. The main psychological symptoms reported were psychological distress after 1 year (65%) and related physical health problems after the death (72%). Depression and mild anxiety were the most reported disorders, with respectively 92.3% and 74.4% of the subjects.

**Conclusion:**

Grief in Lubumbashi is associated with a large number of psychological, social and health problems. Health problems such as gastric is, high blood pressure were often reported. Being accused of witchcraft remains the main social impact. Depression and anxiety were the most psychological problem associated with grief.

## Introduction

Grief is a universal experience faced at one time or another by most people during their lives. Repeatedly, the concept of grief is one of the main sources of psychiatric disorders such as insomnia, loss of appetite, anxiety, and depression [1]. Indeed, some people are susceptible to complications of normal grief. These people are those who are weak in the usual manner or temporarily weakened by the particular circumstances whatsoever on physical health, mental balance and social inclusion are subject to feel hardest traumatic effects of grief [2]. If the duration of grief varies from one individual to another, it's considered that the majority of them are completed after one year. Grief has two main stages, the progress and the work of mourning, bereavement. There are three steps in this period? Of grief: Initial phase or distress phase, which is an impact or stupor phase characterized by a state of shock; central phase called reactive depression or withdrawal. It is the acute period of mourning, an intense look depressed emotional state; the end of mourning or the resolution phase is a phase of recovery, restoration, or adaptation [3]. The role of the grief is to internalize the missing person in itself. This happens by evoking memories of the deceased with other people they knew. The grieving process is a clear expression of the effects of grief work through the pain and movement of psychic regression. It presents mainly in 3 dimensions. The recognition of the reality of the loss is not immediate, and is causing distress and suffering of the bereaved: strengthening internal link with the lost person and taking into account unconscious sense of guilt [3].

Biological aspects of mourning: some endocrine disturbances are often reported such as: a high level of adrenal activity; A response to growth hormone (GH) occurring early in the bereavement and associated non-limiting forms every 2 months [3].

Neurological and biological aspects: the functional magnetic resonance imaging (fMRI) in patients who bereaved photographs and words related to the deceased were shown, with the visual and auditory stimuli given alternately and independently of each other. It appeared that three brain regions were activated: The posterior cingulate cortex, superior and middle frontal gyrus and cerebellum. The first two regions are associated with memory processes (auto biographical memory) related to emotion at the display and recognition of the image. Meanwhile the cerebellum plays a role of coordination and modulation of cognitive functions. The authors also noted a slight activation of the caudate nucleus, a structure that is related to the phenomenon of attachment but they moderated their interpretation due to the lack of a control group of subjects free from grief. These activations are possibly related to the visualization of familiar faces and would need to be explored in case-control studies and on larger groups [4].

## Methods

A cross-sectional study was conducted in Lubumbashi from mars 2012 to September 2012. Lubumbashi is the second largest and the mining city in the Democratic Republic of Congo, with an estimated population: of 1.5 million. A snow ball sampling method was used, which ended up with a sample size of 78 subjects. The snow ball sampling method was used due to social and cultural believes associated with death in Africa. Indeed, it will be more difficult for us to reach people willing to speak openly about their grief experience in Lubumbashi. Two rating tools were used and administered only to respondents who expressed a clear willingness to participate in the study. All municipalities in Lubumbashi (7) were visited and a total of 78 subjects willingly accepted to take part in the study. The Hamilton Anxiety Rating Scale (HARS) and the Beck Depression inventory (BDI) were used. The HARS measures the severity of anxiety and it includes 14 items rated from 0 to 4. The total score is the sum of the scores on each item. It is possible to have two partial scores: a score for psychic anxiety, which is the sum of scores for items 1-2-3-4-5-6-14, and a score for somatic anxiety, which is the sum of scores for items 7 and 13. The BDI is done based on the psychological state of patients in recent weeks, even months. The answers to the questions take into consideration the duration of the state. Depression was considered when the symptoms were present on a daily basis and for at least 3 weeks. Few days of sadness were considered as a situational depression or sadness due to a given situation. It is therefore advisable to wait few weeks before administering the test as there is a good chance that the symptoms may have disappeared by itself in situational depression.

## Results

Our study included 78 bereaved. The majority of subject who reported complicated bereavement (87.2%) were below 50 years. There were more women compared to males (65.4%). most of the subjects reporting complicated bereavement were singles 39 (50%). It was also reported that about half of those reporting complicated bereavement (48.7%) were either jobless or with a liberal profession, such as small traders, merchants, artisans. None of the respondent was reported to have been diagnosed or to have presented any type of psychiatric disorders in their medical history (Table 1). Complicated bereavement occurred in almost 90% of the subjects. Indeed, 70 (89.74%) reported to have a great sadness resulting from the fact that they have seen their loved one suffering and dying within a month. A large proportion of the bereaved (73.08%) received social support. The main problem which was reported to disrupt the smooth running of the bereavement was being accused of sorcery; of being accused directly responsible of the death was reported by 22 individuals 41,5%. Almost 80% (62 subjects) reported pathologic bereavement as a result of a complicated bereavement lasting more than a year after the loss of the loved ones, among which 51 (64,5%) of the bereaved reported persistent complication. Among reported somatic symptoms, epigastric pain 26 (46,4%) was the most frequent, followed by withdrawal 16 (28,6%) 6%) and hypertension11 (19,6%) (Table 2). Using the HARS and BDI scales for depression and anxiety, the majority of bereaved who reported anxiety and depressive symptoms were female 51 (65.4%) (Table 3).

**Table 1:** Socio-demographic parameters (Age, Sex, Marital Status and Occupation)

	*Effective (N = 78)*	*Percent*
***Age***		
*≤ 50 years*	68	87,2
*≥ 51 years*	10	12,8
***Sex***		
*Female*	51	65,4
*Male*	27	34,6
***Civil Status***		
*Single*	39	50,0
*Married*	6	7,7
*Widow (widows)*	33	42,3
***Occupation***		
*Employee*	10	12,9
*Unemployed*	14	17,9
*Liberal*	22	28,2
*Student*	16	41,0

**Table 2:** Complications of normal grief

	*Effective (N = 78)*	*Percent*
***How long has he suffered?***		
*< 1 month*	70	89,74
*> 1 month*	8	10,26
***Have you received counseling for grief?***		
*Yes*	57	73,08
*No*	21	26,92
***Type problems***		
*Disputes turn the deceased's property*	17	32,1
*Rejected by family*	14	26,4
***How long have you suffered from his absence***		
*< one year*	16	20,5
*> one year*	11	14,1
*Always*	51	65,4
***Type healthproblems***		
*evils’ Stomach*	26	46,4
*Withdrawal*	16	28,6
*Hypertenssion*	11	19,6
*Hypertenssion and Withdrawal*	3	5,4

**Table 3:** Complications of normal grief sex and depression scale and Hamilton also anxiety scale of Hamilton

	*Rating Scale Hamilton Depression*			
sex	**Mild depressive symptoms**	**Mild to moderate depressive symptoms**	**Moderate to severe depressive symptoms**	**Total**
*female*	45	6	0	1
	(57,6%)	(7,7%)	0%	65,40%
male	27	0	0	7
	-34,60%	0%	0%	34,60%
TOTAL	72	6	0	8
	(92,3%)	(7,7%)	0%	00,00%
	*Evaluation scale Hamilton anxiety*			
sex	**Light**	**Mild to moderate**	**Mild to severe**	**Total**
*female*	33	18	0	1
			0%	65,40%
male	25	2	0	7
			0%	34,60%
TOTAL	58 (74,4%)	20 (25,6%)	0	8
			0%	00,00%

## Discussion

Simon NM during his clinical observations describes the complications of mourning as a post-traumatic stress disorder, which occurs in 7% of bereaved persons [5]. Our findings showed that 92.3% of the subjects reported anxio-depressive symptoms. The bereaved person inflicts in our social community a double shock, firstly the loss of loved people and secondly social issues insecurity in which she goes through during several months. This shows anxio depressive state during morning ritual. Chiu YW study on cancer patients shows that the important determinants of bereavement were female gender, parent-child relationships, religion, and a medical history of mood disorder [6]. In line with Chiu, our findings also demonstrate that the majority of affected subjects were females followed by singles, parents and widows. That's explained by the fact that a woman in our social community doesn't work, so the loss of spouse constitutes the beginning of socio economical problems that are accentuated by confiscation of all the properties by the family members of the defunct. Bryant RA reported in his study conducted in Australia that 10-15% of bereaved presented severe complications assimilated to a pathological bereavement but significantly different from depression and anxiety, thus expanding the scope of psychiatric morbidity [7]. In contrast, our study showed that the majority of subjects reported both somatic and psychological symptoms, with depression and anxiety being the most frequent. Half of those reporting somatic symptoms also had psychological problems. In our social community, socialization of mourning has a positive side in sense that constitutes a real psychological support of the bereaved person. He doesn't feel lonely that's to say during the process mourning time, people has more physical (body) problems like gastric, chiefly caused by lack of food adequate food, and rarely psychic problems to the support of brothers and friends.

Prigerson studied 291 bereaved in Massachusetts, USA and reported that distress and dysfunction resulting from the loss of a loved one can be the basis for an extension of mourning, which is an evident complication [8]. Our study found that 68% of the subjects saw their course of mourning disturbed by different problems, such as lack of family supports, rejection in the street immediately after the burial of the deceased, or being treated as a which craft and therefore the direct cause of death. Carnassir et al, in their clinical study have shown that bipolar disorder may have symptoms exacerbated by a commemoration of mourning, displaying therefore all the symptoms of bereavement [9]. In our study during the commemoration of mourning is more somatic symptoms that is followed anxiety and depressive disoders. Klinitzke's study on patients in hospital environment demonstrated that 14.2% of patients with depressive disorder were bereaved within 12 months. He also found that there was a correlation with the level of education [10]. Our findings showed that age female gender and unemployment were correlated with depressive symptoms. In our study, 14.1% of respondents said having suffered more than a year. Most of the times, somatic symptoms as at the level of education, klinetzke confirms our results in his study. Hinton highlighted in his study on the survivor of the Pol pot genocide in Cambodia that there is a correlation between post-traumatic stress disorder and the complicated grief syndrome. He also found that the cultural context of an unworthy death was not followed by a ritual or a funeral. Besides, 76% of the reported symptoms were bad dreams [11]. Maccalum in his meta-analysis considered that prolonged mourning/bereavement should be understood as a complication of bereavement, and approximately 10% of the bereaved develops it [12]. It has been difficult to establish in our social community a clear difference between prolonged grief and complicated grief in the sense that over 70% of respondents claimed to suffer a long time during the loss of their loved ones, economic and social problems, insufficient support from the entourage comes into account as contributing factors. Mallinsao in his meta-analysis demonstrates that grief is a universal response after the passing of a loved one, and normally the symptoms will subside naturally for a while. However, in people with HIV, complications are more severe, accompanied by physical alternation and suicide attempts, hence the importance of a close therapeutic counselling and the cognitive behavioural therapy [10]. This corroborate the need and importance of offering psychological and psychiatric support to bereaved subject in Lubumbashi, DR Congo as the cultural and social context play a major role in complicated bereavement.

## Conclusion

Our findings also suggested that grief is actually a universal answer to the loss of a loved one. However, in this setting factors such as age, gender, socio-cultural believes related to the inheritance, rejection from the family and the society were considered as major risk factors to complicated bereavement. This stresses the importance of taking these factors into consideration and of offering proper management of bereavement by psychiatrists? From the announcement of the death until the resolution of the grief. Mental health care professionals / psychiatrists should be involved in the management of complicated bereavement to prevent mental health problems and contribute to the mental well-being of the community.

### What is known about this topic

- Grief is a psychological reaction due to the loss of a loved one, each person there is confronted.
- Mourning (grief) has 3 phases, that distress followed the decline (withdrawal) and finally a resolution.
- The grieving process is expressed through the work of mourning (grief) by the moral suffering and psychic retrogression.

### What this study adds

- Women and the unemployed men are more affected by complications of death (grief) in our midst.
- Death in our community most cases as a cause of witchcraft and several people incriminated.
- The epigastric pain and high blood pressure are the main somatic complication while anxio depressive crises they are the psychic complication in our midst.
